# Youssef syndrome

**DOI:** 10.1007/s00404-023-07358-1

**Published:** 2024-01-29

**Authors:** Christl Reisenauer, Bastian Amend

**Affiliations:** 1 Department of Gynecology and Obstetrics, University Hospital Tübingen, Calwerstrasse 7, 72076 Tübingen, Germany; 2 Department of Urology, University Hospital Tübingen, Hoppe-Seyler-Str. 3, 72076 Tübingen, Germany

## Presentation

A 35-year-old healthy woman presented with amenorrhea and cyclical haematuria (menouria) at the expected time of menses. She was continent, with no loss of urine at any time. The symptoms began after her first caesarean delivery due to placenta praevia three years previously. Intraoperatively a small bladder injury occurred followed by a 7-day continuous bladder drainage. 

A cystoscopy was performed by a urologist and a vesical endometriosis was diagnosed clinically and histiopathologically. 6 months after unsuccessful hormonal therapy (Visanne®), she was referred to our hospital.

**Fig. 1:**
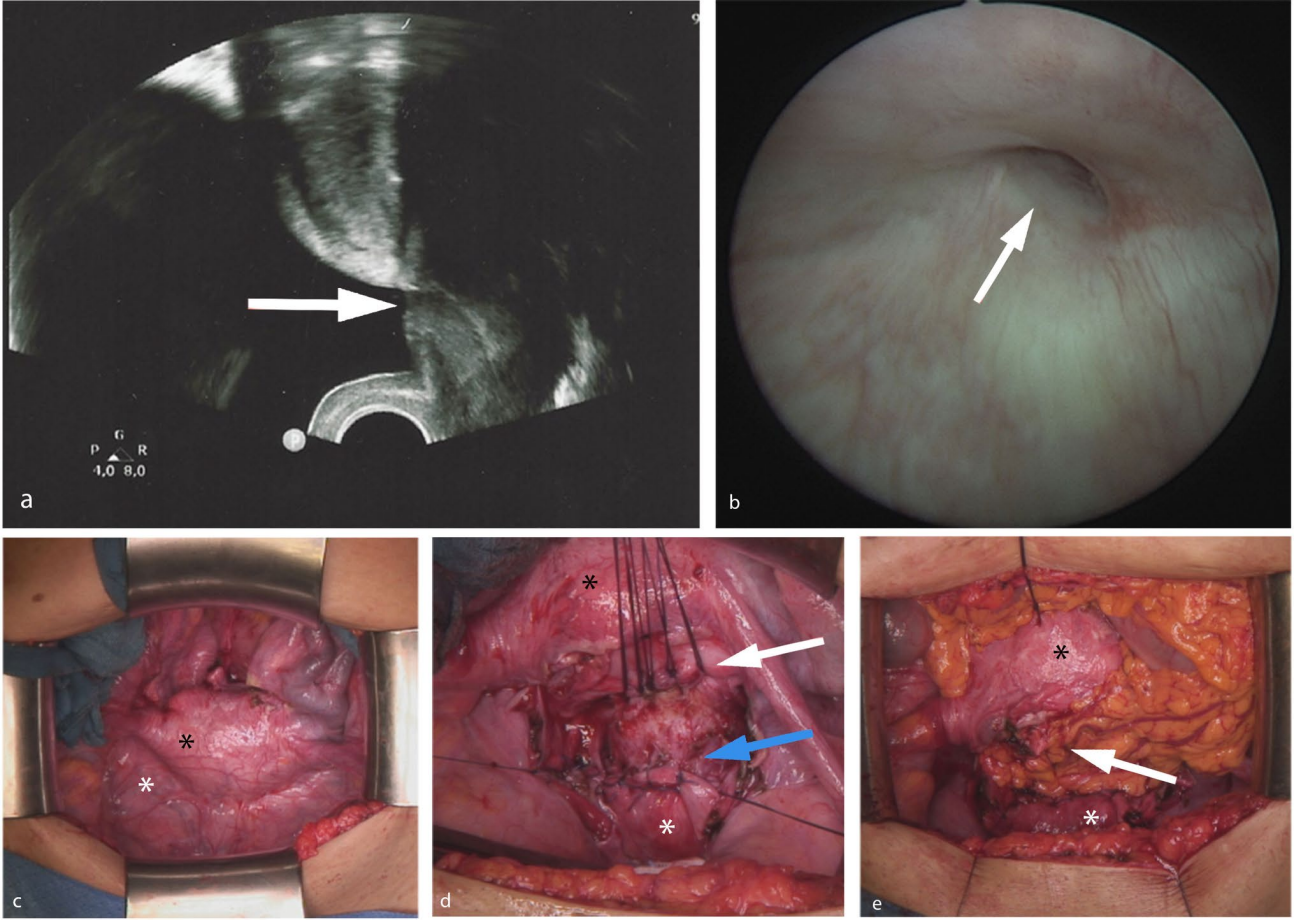
**a** Pelvic floor sonography: fistulous tract between the posterior bladder wall and the isthmus uteri (white arrow); **b** cystoscopy: fistula opening (white arrow); **c** laparotomy: uterus (black asterisk), bladder (white asterisk), **d** the fistula openings were closed (uterus suture-line white arrow, bladder suture-line blue arrow), **e** omental flap interposition between the uterus and the bladder (white arrow)

The pelvic floor sonography revealed a fistulous tract between the posterior bladder wall and the isthmus uteri (Fig. 1a). Cystoscopy found a 1.5-cm diameter fistula in the right supratrigonal area (Fig. 1b). Hysteroscopy was not possible to be performed due to cervical stenosis. The cervical stenosis explained the woman’s urinary continence.

The utero-vesical fistula surgery was performed abdominally (Fig. 1c). After separation of the bladder from the uterus followed by freshening of the edges, a mechanical dilatation of the cervix, from the internal to the external os, was performed using Hegar dilatators. The fistula openings were then each closed with two tension-free layers (Fig. 1d). Between the uterus and the bladder an omental flap was interpositioned to improve the chance of a good outcome (Fig. 1e). The bladder was drained by a suprapubic catheter for three weeks. The postoperative period was uneventful and the woman remained asymptomatic with resumption of normal menses and no clinical evidence of fistula recurrence at two years postoperative review.

## Discussion

The Youssef syndrome (amenorrhea, cyclical haematuria and urinary continence) [1] is one of the possible long-term morbidities secondary to caesarean sections. Physicians may easily be misled and diagnosis delayed. Due to a continuous increase in caesarean section rates worldwide, this type of vesico-uterine fistula may be encountered more frequently. Gynecologists and urologists should be aware of its clinical presentation.

## Data Availability

Not applicable.
